# The Implementation of the Biopsychosocial Model: Individuals With Alcohol Use Disorder and Post‐Traumatic Stress Disorder

**DOI:** 10.1002/brb3.70230

**Published:** 2024-12-31

**Authors:** Fernando Hinostroza, Michele M. Mahr

**Affiliations:** ^1^ Centro de Investigación de Estudios Avanzados del Maule, Vicerrectoría de Investigación y Postgrado Universidad Católica del Maule Talca Chile; ^2^ Centro de Investigación en Neuropsicología y Neurociencias Cognitivas, Facultad de Ciencias de la Salud Universidad Católica del Maule Talca Chile; ^3^ Escuela de Química y Farmacia, Departamento de Medicina Traslacional, Facultad de Medicina Universidad Católica del Maule Talca Chile; ^4^ Centro para la Investigación Traslacional en Neurofarmacología Universidad de Valparaíso Valparaíso Chile; ^5^ Rehabilitation Psychology, Health Science Center Texas Tech University Lubbock Texas USA

## Abstract

**Introduction:**

This extensive literature review investigates the relationship between post‐traumatic stress disorder (PTSD) and alcohol use disorder (AUD), focusing on the neurobiological changes associated with their co‐occurrence. Given that these disorders frequently coexist, we analyze mechanisms through which alcohol serves as a coping strategy for PTSD symptoms, particularly highlighting the drinking‐to‐cope self‐medication model, which suggests that alcohol use exacerbates PTSD symptoms and complicates recovery.

**Methods:**

A systematic literature search was conducted across multiple databases, including PubMed and Google Scholar, to identify studies examining the intersection of the biopsychosocial model with PTSD, AUD, and associated neural alterations.

**Results:**

Findings demonstrate that chronic PTSD is associated with progressive dysfunction in the amygdala, hippocampus, prefrontal cortex, hypothalamic–pituitary–adrenal axis, and white matter pathways. Also, our findings underscore alterations within the reward system, prefrontal cortex, hippocampus, amygdala, basal ganglia, and hypothalamic–pituitary–adrenal axis that contribute to the pathophysiology of AUD. Our results support the notion that a biopsychosocial framework is essential for contemporary addiction treatment, particularly in the context of alcohol addiction and PTSD.

**Conclusion:**

PTSD frequently leads individuals to use alcohol as a maladaptive coping strategy, ultimately resulting in neuroadaptive alterations across critical brain regions. These neurobiological changes contribute to the development and maintenance of AUD. The findings reiterate the necessity of employing a biopsychosocial model in treating individuals grappling with both PTSD and AUD. This model allows for a comprehensive understanding of the unique challenges faced by this population, integrating biological, psychological, and social factors that influence recovery.

## Graphical abstract

Post‐traumatic stress disorder and alcohol use disorder are debilitating conditions that lead to negative outcomes. The authors explore the intricate relationship between conditions and significant neurobiological alterations that may impact an individual's behaviors, treatment plans, and overall lifestyle.

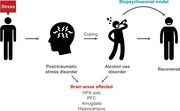

## 1 Introduction

There are several factors to consider when addressing and implementing treatment plans for individuals with alcohol use disorder (AUD) and post‐traumatic stress disorder (PTSD). Both of these psychiatric conditions are listed within the *Diagnostic and Statistical Manual of Mental Disorders* (DSM‐V‐TR), with specific criteria for clinicians to refer to when diagnosing individuals during the evaluation process. This paper provides an in‐depth overview of how the neurological pathways are altered as a result of using alcohol to cope with PTSD. Within the literature, there have been numerous etiological models that describe the relationship between PTSD and AUD, including shared genetic risk and phenotypic causality; however, the most recognized model of etiologic correlation is the drinking‐to‐cope self‐medication model (Hawn, Cusack, and Amstadter 2020).

Several studies have revealed that participants in their study concluded that an increase in alcohol use was connected to subsequent greater PTSD symptoms, suggesting that alcohol use may impact the maintenance of PTSD symptoms as well as stifle PTSD recovery (Tripp et al. 2020; Ben‐Toloyai et al. 2024). Treatment protocol and the process may vary based on the individual's needs, resources, and severity of the condition. The following paper provides suggestions on potentially effective treatment modalities for both AUD and PTSD through a neuroscience lens. A primary focus of the biopsychosocial model is treatment modalities that may be viable, effective, and specific to assisting individuals with both PTSD and AUD.

The etiology of chronic conditions or illnesses stems from several factors, and it would be naive to assume that there is only one cause for disease based on a health psychology viewpoint (Frazier 2020). The interaction of biopsychosocial factors contributes to the pathways of onset, manifestations, and treatment for physical and mental health conditions (Frazier 2020). When applying this model, health‐care clinicians assisting individuals with AUD and PTSD can target specific areas that require perhaps more extensive treatment, assessment, resources, or support based on a comprehensive plan. Finally, more current research on the biopsychosocial model has included current social determinant factors considered relevant when treating health conditions. For example, potential epidemiological studies assume that the risks for several illnesses, physical and mental, begin in development, some in childhood and that these risks contain social factors such as poverty, social isolation, family, neglect and abuse, and lifestyle components such as nutrition and physical activity (Bolton and Gillett 2019). The purpose of this extensive literature review is to provide a detailed examination of the connection between PTSD, AUD, and the neurological alterations when an individual is diagnosed with AUD.

## 2 Methods

We conducted a systematic literature search across multiple databases, including PubMed and Google Scholar. The search strategy was designed to capture a comprehensive selection of studies relevant to the biopsychosocial model's application to brain function, PTSD, and alcohol addiction. To develop a precise set of search terms, we combined variations of the “biopsychosocial model” with keywords such as “brain,” “PTSD,” “alcohol addiction,” and related terms. Boolean operators and tailored combinations (e.g., “brain AND PTSD,” “biopsychosocial model AND alcohol addiction”) were applied to optimize search specificity and reduce irrelevant results. Inclusion criteria were restricted to peer‐reviewed articles available in English, spanning from the inception of each database to December 2023.

## 3 Post‐Traumatic Stress Disorder

PTSD is a consequence of individuals who have experienced events that suggest fear, helplessness, or terror (Patock‐Peckham et al. 2020). This condition is defined as a psychiatric disorder that develops in the months and years as a consequence of exposure to extensive trauma (Ressler et al. 2022). PTSD is not limited to one type of trauma and may include natural disasters, violence or abuse (criminal or familial), hostage‐taking, or life‐threatening conditions. To combat the psychological and physiological repercussions of this debilitating diagnosis, individuals may drink alcohol excessively. There has been a considerable amount of research evidence that suggests a conceptual framework in which PTSD can be partially viewed as a condition of fear dysregulation, leading to the study of neuroscience approaches for this concern, which offers opportunities to advance the field through translational neuroscience approaches (Ressler et al. 2022). The World Health Organization (WHO) World Mental Health Surveys recently predicted that the lifetime prevalence of PTSD was 3.9% across nations and 5.6% of individuals who have been exposed to trauma (Koenen et al. 2017).

In regard to the neurobiology of PTSD, numerous brain areas are assumed to be correlated to the pathophysiology of PTSD, including the hippocampus, amygdala, and cingulate, alongside the medial and dorsolateral prefrontal cortex (PFC) (Dell'Osso et al. 2009). Research studies are similar in that individuals with PTSD suffer from an abundance of fear memories and extinction learning (Takei et al. 2011). Another recent study by Kim, Lee, and Yoon (2020) about the inflammatory markers related to PTSD and neuroimaging showed that there was a reduction in cortical thickness within regions of the PFC and hippocampal volume and a potential correlation between amygdala activity and systemic inflammation for participants experiencing PTSD in their review.

Progressive treatment approaches for PTSD may essentially be facilitated by a more in‐depth conceptualization of how the brain function alters when symptoms improve (Garrett et al. 2019). Patel et al. (2012) note that neuroimaging studies of adults with PTSD support a neurological approach model, often noting that PTSD is correlated with aberrant structure and function altercations of these brain regions.

## 4 Alcohol Use Disorder

AUD is a gradual and chronic condition characterized by compulsive and uncontrolled drinking despite the negative consequences. The *Diagnostic and Statistical Manual of Mental Disorders 5th Edition* (DSM‐V) defines it as a maladaptive pattern of substance use causing severe distress and impairment. It can be cataloged as mild, moderate, or severe. It involves alcohol desire, craving, frequent alcohol use, tolerance, and withdrawal symptoms such as vomiting, anxiety, sweating, auditory and visual hallucinations, and shakiness (National Institute for Health and Care Excellence 2017).

The National Survey on Drug Use and Health estimates that 29.5 million people suffered AUD in 2021 (Substance Abuse and Mental Health Services Administration 2021). In addition, AUD negatively impacts individuals, friends, relatives, and work. Only for the United States does it cost $249 billion per year (Sacks et al. 2015). Worldwide, alcohol consumption causes 5.3% of deaths per year, ∼3 million, according to the WHO. Moreover, 13.5% of deaths in people 20–39 years old are related to alcohol use.

AUD is an avoidable and curable condition (Fairbanks et al. 2020). Because the etiology of AUD stems from complicated and synergistic neurobiological, genetic, and environmental factors, there are many treatment plans that are effective for everyone (Knox et al. 2019). Rogal et al. (2020) suggest that AUD is undertreated in its entirety, while pharmacotherapy is specifically underutilized.

## 5 Dual Diagnosis of PTSD and AUD

PTSD and AUD co‐occur often and are correlated with several unhealthy and poor outcomes (Hawn, Cusack, and Amstadter 2020). The negative outcome for individuals with comorbid AUD and PTSD is worse than those who have a solo diagnosis for each disorder without comorbidity (McCarthy et al. 2010). Previous trauma, mistreatment, neglect, and successive adversity are psychosocial stress factors that have been positively correlated with the misuse of alcohol, the progression of AUD, and a decrease in recovery (Guinle and Sinha 2020). Furthermore, literature in alcohol‐related research has documented that individuals with anxiety disorders who stated that they use drinking to cope with their anxiety symptoms had higher levels of drinking and higher rates of DSM‐IV alcohol dependence than those who did not report drinking to decrease their symptoms of anxiety (Menary et al. 2011).

Several hypotheses attempt to explain the correlation of PTSD with AUD. For example, some research suggests that this is similar to the learning theory, where alcohol use is being ingested to decrease or eliminate the symptoms associated with PTSD temporarily (Hawn, Cusack, and Amstadter 2020). However, in a recent article by Hawn, Cusack, and Amstadter (2020), the scholars note that the addiction and mental health field often assume that individuals with PTSD are engaging in substances or alcohol to deal with their PTSD symptoms; however, individuals with PTSD may be drinking to cope with other traits that are characterized by negative intrinsic experiences, such as depression and anxiety. It is important to recognize that individuals with PTSD may have other mental health concerns, and self‐medicating with substances appears to be the coping mechanism to deal with the symptoms of the condition. Moreover, the correlations between PTSD, AUD, and their comorbidity on functional consequences, such as sleep, anger, and occupational stress, are noted in the literature (Zegel et al. 2022). It would be advantageous for health professionals to examine the relationship between wellness factors such as those listed above in conjunction with PTSD and AUD.

## 6 Neurobiological Basis of PTSD

Chronic PTSD has been associated with progressive deterioration of function within specific brain structures. One proposed neural‐circuit model of PTSD highlights the functional relationships among critical brain structures: the amygdala, hippocampus, PFC, and the hypothalamic–pituitary–adrenal (HPA) axis (Bremner et al. 1995, 2003; Hayes, Hayes, and Mikedis 2012; Heyn et al. 2019). These structures are essential in fear processing and emotional regulation.

### 6.1 Amygdala Dysfunction

The amygdala is a brain structure adjacent to the hippocampus involved in fear learning, emotion, and reward processing (Cheng et al. 2003, 2006). It has three functional regions: the medial, basolateral, and central nuclei, collectively contributing to emotional responses. In PTSD, the amygdala exhibits abnormal activation and hyperactivity, which relates to alterations in fear learning (Blechert et al. 2007; Hayes, Hayes, and Mikedis 2012; Jovanovic et al. 2010; Patel et al. 2012). In non‐pathological conditions, the medial PFC (mPFC) connects to and inhibits the amygdala, a process known as top‐down cortical inhibition. However, in this pathology, the hypoactivation of the mPFC disrupts this inhibitory control, leading to increased amygdala activity (Hayes, Hayes, and Mikedis 2012; Patel et al. 2012).

The consequences of amygdala hyperactivity extend beyond alterations in fear learning. This dysregulation significantly contributes to heightened fear responses and a pervasive state of hypervigilance in individuals with PTSD (Milad et al. 2009). Furthermore, increased amygdala activation is implicated in difficulties regulating emotions, resulting in symptoms such as intensified fear associations, heightened anxiety, intrusive thoughts, and persistent hyperarousal.

### 6.2 Hippocampal Alterations

The hippocampus, a critical structure for fear learning, declarative memory, retrieval, and spatial navigation, is significantly impacted in PTSD patients. Foremost, there is a consistent observation of reduced hippocampal volume attributed to prolonged exposure to elevated cortisol levels resulting from chronic stress and trauma exposure (Bremner et al. 1995; Carrion et al. 2010). This structural modification, coupled with diminished hippocampal activity, contributes to the intricate cognitive and emotional impairments observed in PTSD.

Neuroimaging studies have underscored the pivotal role of the hippocampus in fear learning, unveiling reduced hippocampal activation in PTSD individuals during such processes (Milad et al. 2009). The diminished activation is intricately linked to the impaired ability to contextualize and process traumatic memories. The hippocampus, typically engaged in creating contextual associations, fails to effectively integrate traumatic experiences into a broader cognitive framework, leading to the persistence of vivid and emotionally charged traumatic memories.

Evidence suggests a negative correlation between hippocampal volume and the severity of PTSD symptoms. Gilbertson et al. (2002) demonstrated that a smaller hippocampal volume is associated with more pronounced PTSD symptoms. This correlation further underscores the significance of hippocampal alterations in shaping the clinical manifestation and severity of PTSD.

### 6.3 The PFC Dysregulation

The PFC is the most anterior part of our brain. It is a pivotal region for higher order cognitive functions. It orchestrates decision‐making, problem‐solving, and impulse control (Barraclough, Conroy, and Lee 2004; Boes et al. 2009; Mushiake et al. 2009). The PFC is not an isolated entity but rather intricately connected to various brain regions through functional and structural connectivity. Functional connectivity refers to the coordination and synchronization of neural activity between different brain regions, even when they are anatomically distant. Structural connectivity applies to white matter connecting different brain regions. The PFC receives direct inputs from crucial regions like the amygdala, hippocampus, thalamus, basal ganglia, ventral tegmental area (VTA), cerebellum, hypothalamus, sensory cortices (i.e., visual, somatosensory, auditory, and olfactory cortices), and from other regions of the PFC. Also, the PFC sends long projections to the amygdala, hippocampus, basal ganglia, thalamus, anterior cingulate cortex, orbitofrontal cortex, parahippocampus, Broca's area, and dorsolateral and ventromedial prefrontal cortices (Haber et al. 2022; Le Reste et al. 2016). This extensive connectivity positions the PFC as a central hub in integrating information and generating specific behavioral responses.

The ventral region of the PFC has been associated with emotional responses, including conditioned fear memory and anxiety (Cho, Deisseroth, and Bolshakov 2013; Sierra‐Mercado, Padilla‐Coreano, and Quirk 2011). In individuals with PTSD, the ventromedial PFC (vmPFC) volume is reduced (Heyn et al. 2019). The alteration in the PFC leads to an inhibitory impairment resulting in amygdala overactivation (Hayes, Hayes, and Mikedis 2012; Patel et al. 2012). Additionally, the dorsolateral PFC (dlPFC), responsible for executive functions, exhibits altered activation patterns. Reduced dlPFC activation is observed during emotional anticipation, indicating potential deficits in executive functions and impulse control (Aupperle et al. 2012; Holmes et al. 2018). Conversely, an increased dlPFC activation during the fear acquisition strengthens fear‐association formation, rendering them more resilient to extinction (Milad et al. 2009; Rougemont‐Bucking et al. 2011).

### 6.4 HPA Axis Dysregulation

The HPA axis governs the body's response to stress, including releasing stress hormones such as cortisol. The hypothalamic paraventricular nucleus releases the corticotropin‐releasing hormone (CRH) in the external part of the median eminence to enter the hypothalamic–hypophyseal portal system (Bloom et al. 1982). Thus, the CRH reaches the adenohypophysis and stimulates the corticotroph cells to release the corticotropin hormone (ACTH) (Bruhn, Plotsky, and Vale 1984). The ACTH enters the blood system and goes to the adrenal gland to induce the production and release of cortisol. The produced cortisol regulates its release through a negative feedback loop to maintain homeostasis.

In PTSD individuals, the HPA axis exhibits dysregulation, marked by an exaggerated response and increasing cortisol secretion (Bremner et al. 2003). This heightened HPA axis activity is thought to stem from the persistent perception of threat associated with traumatic memories. Consequently, individuals with PTSD may endure a state of chronic hyperarousal characterized by symptoms such as hypervigilance, exaggerated startle responses, and increased anxiety (Grillon et al. 2009; Woodward, Murburg, and Bliwise 2000).

The dysregulation of the HPA axis in PTSD has profound implications for the severity of the disorder. While a healthy stress response involves an elevation in the levels of cortisol in response to a stressor, followed by a negative feedback mechanism that signals the HPA axis to reduce cortisol production to restore homeostasis, in PTSD, these feedback mechanisms can become impaired. This results in prolonged elevation of cortisol levels without an immediate threat. This sustained hyperactivity of the HPA axis can have detrimental effects on the brain and the body, contributing to symptoms such as memory problems, emotional dysregulation, and physical health issues, exacerbating the overall severity and chronicity of PTSD. Recently, new drugs, such as diosgenin and geraniol, have been tested in mouse models of PTSD, showing that both positively modulate the HPA, increase levels of serotonin and dopamine (DA), and reduce oxidation and inflammation (Ben‐Azu et al. 2023, Ben‐Azu et al. 2024). Thus, promising new pharmacological approaches.

### 6.5 White Matter Abnormalities

Individuals with PTSD manifest discernible alterations in white matter integrity. Utilizing diffusion tensor imaging (DTI), Ge et al. (2023) showed that PTSD patients exhibit diminished fractional anisotropy (FA) in the body and the splenium of the corpus callosum, the corticospinal tract, the fronto‐occipital fasciculus, and the superior and inferior longitudinal fasciculus, indicating white matter structural abnormalities. The splenium of the corpus callosum, connecting the temporal and occipital regions of both hemispheres, plays a crucial role in interhemispheric communication (Blaauw and Meiners 2020). The corticospinal tract, which comprises axons from the cerebral cortex to the spinal cord motor neurons, is implicated in motor control (Welniarz, Dusart, and Roze 2017). The superior longitudinal fasciculus, linking the frontal, parietal, and temporal regions, has been associated with attention and memory processes (Kamali et al. 2014; Kawamura and Naito 1984). Furthermore, the inferior longitudinal fasciculus connects anterior temporal areas to occipital and temporal‐occipital areas, facilitating the integration of visual and emotional information (Herbet, Zemmoura, and Duffau 2018).

In addition to diminished FA, there is evidence of increased FA in PTSD individuals within the anterior thalamic radiation and the uncinate fasciculus (Zhang et al. 2023). The anterior thalamic radiation, connecting the anterior thalamic nuclei with the PFC (Coenen et al. 2012), is implicated in emotional expression (Spalletta et al. 2013). Augmented FA in this pathway may be associated with persistent fear responses (Yin et al. 2011). The uncinate fasciculus, linking the anterior temporal region with the orbitofrontal region, ventromedial frontal cortex, and the hippocampus, amygdala, and temporoparietal cortex, is vital for episodic memory formation and the regulation of emotional responses (Catani et al. 2002; Von Der Heide et al. 2013). Consequently, the structural alterations in the uncinate fasciculus among PTSD patients may contribute to deficits in processing social‐emotional stimuli.

## 7 Neurobiological Basis of AUD

AUD is characterized by a cycle consisting of preoccupation and anticipation, binge and intoxication, and withdrawal and adverse effects stages related to different cerebral structures such as the PFC, basal ganglia, and amygdala, respectively (Yang et al. 2022) and the reward system (Koob et al. 1998).

### 7.1 Reward System Pathway

A central aspect of AUD involves alterations in the dopaminergic pathways within the brain, particularly in the striatum, a critical region implicated in reward processing and habit formation.

The mesolimbic pathway, which originates from the VTA and extends to the nucleus accumbens (NAc) in the striatum, is crucial in mediating the rewarding effects of alcohol. Chronic alcohol exposure induces neuroadaptive changes in this pathway, leading to dysregulation of DA release and subsequent reward processing. Alcohol increases DA release from VTA dopaminergic neurons onto the NAc neurons (Koob et al. 1998). The rewarding effect is obtained by the fast release of large amounts of DA that activate the excitatory DA D1 receptors (Caine et al. 2007). Thus, alcohol induces a similar state produced by the phasic firing of dopaminergic neurons related to rewarding stimuli (Covey, Roitman, and Garris 2014).

Additionally, the dorsal striatum, encompassing the caudate nucleus and putamen, plays a pivotal role in habit formation and motor control. Recent research has highlighted the involvement of the dorsal striatum in the development of compulsive alcohol‐seeking behavior (Everitt and Robbins 2016). Neuroimaging studies have demonstrated alterations in striatal activation in individuals with AUD during cue‐induced craving (Vollstadt‐Klein et al. 2011). These findings suggest a shift toward habitual, compulsive alcohol‐related behavior mediated by striatal dysfunction.

### 7.2 AUD and PFC

In the context of addictions, such as AUD, the PFC plays a pivotal role, particularly in the preoccupation and anticipation stage associated with the resurgence of drug‐seeking behavior following abstinence. Structural alterations in the PFC are evident in individuals with AUD, manifesting as a reduction in gray matter volume in key regions, including the mPFC and the right lateral PFC (lPFC). Studies have linked these structural changes to a heightened risk of alcohol relapse (Fein et al. 2002; Medina et al. 2008; Rando et al. 2011; Rosenthal et al. 2019; Wang et al. 2016). Notably, the degree of gray matter reduction in the dlPFC is closely associated with the duration of an individual's alcohol consumption (Fein et al. 2002; Fortier et al. 2011).

These structural alterations are not merely anatomical but are functionally significant, contributing to cognitive deficits, impaired decision‐making, and diminished inhibitory control. The consequence is impulsive behavior and poor judgment concerning alcohol consumption. Functionally, the PFC shows reduced connectivity with the cerebellum, impacting the thalamocortical cerebellar pathway in AUD (Rogers et al. 2012). Furthermore, during tasks requiring executive control, there is a notable reduction in the activation of the dlPFC (Pfefferbaum et al. 2001).

Alcohol consumption not only impacts the PFC function but also disrupts its connectivity with corticostriatal pathways related to motivation (Robinson and Berridge 1993). Also, in AUD patients, exposure to either stressful stimuli or alcohol‐related cues leads to diminished activity in the vmPFC, a region crucial for emotional regulation. This change might contribute to heightened urges for alcohol consumption and a heightened risk of relapse (Seo et al. 2013).

### 7.3 Hippocampus

In individuals diagnosed with AUD, compelling evidence underscores the consequences of prolonged alcohol consumption on the hippocampus, a region crucial for memory and cognitive functions. Specifically, adolescents with AUD exhibit a notable reduction in left hippocampal volume (Nagel et al. 2005). This finding is indicative of the early and detrimental effects of alcohol on brain structures during critical developmental periods.

Further insight into the nuanced alterations within the hippocampus comes from studies focusing on specific subfields. Investigations by Zahr et al. (2019) have revealed smaller CA2 and CA3 regions in individuals with AUD. Strikingly, these reductions do not align with age in comparison to control subjects, suggesting a unique vulnerability of these subregions to the effects of alcohol.

### 7.4 Amygdala

The intricate relationship between the amygdala and AUD unveils a critical nexus in understanding the multifaceted aspects of addiction. Primarily, the modulation of the amygdala by the PFC has emerged as a critical factor in alcohol craving and relapse, especially in the context of stress (Koob and Volkow 2016).

The amygdala's pivotal role in the withdrawal and negative affect stage of AUD amplifies the emotional toll associated with addiction. During withdrawal, heightened anxiety and stress ensue, leading to a cascade of negative emotions, including malaise, dysphoria, emotional pain, and diminished interest in natural rewards. To alleviate these distressing symptoms, individuals often resort to increased drug consumption, perpetuating the addictive cycle. Notably, AUD patients exhibit a reduction in amygdala volume during this stage, a phenomenon intricately linked to negative urgency and heightened anxiety levels (Tomasi et al. 2021). Furthermore, a smaller amygdala has been correlated with increased alcohol craving and a heightened probability of relapse, underlining the pivotal role of this structure in reinforcing addictive behaviors (Wrase et al. 2008).

Beyond the traditional amygdala, the extended amygdala (EA), which comprises the NAc shell, the central nucleus of the amygdala, and the bed nucleus of the stria terminalis, adds another layer of complexity. Operating in the negative affect stage of addiction, the EA is implicated in fear conditioning and the emotional element of pain processing (LeDoux 2000; Neugebauer et al. 2004). In AUD, abstinent patients manifest increased activation of the EA when they are exposed to alcohol cues, underscoring the heightened emotional response to environmental triggers (Schneider et al. 2001).

### 7.5 Basal Ganglia

The basal ganglia, encompassing interconnected structures such as the striatum (caudate nucleus and putamen), globus pallidus, subthalamic nucleus, and substantia nigra, plays a pivotal role in motor control and reward processing. These critical structures are negatively affected by AUD, contributing to the intricate neurobiological landscape of addiction (Koob and Volkow 2016).

Structural alterations within the basal ganglia have been observed in individuals with AUD, with studies revealing a significant reduction in volume, particularly in the caudate nucleus and putamen (Tomasi et al. 2021). These volumetric changes have been associated with the motor dysfunction commonly seen in AUD, including impaired coordination and fine motor skills, highlighting the widespread impact of alcohol on the neural substrates governing movement (Sullivan 2003).

The habitual seeking and consumption of alcohol, a hallmark of AUD, involve intricate basal ganglia circuitry. Specifically, activating the striato‐pallido‐thalamo‐cortical circuit has been identified as a critical player in habit formation (Belin et al. 2009). This circuitry forms the neural basis for the learned behaviors associated with repeated alcohol consumption. Moreover, the PFC sends excitatory projections to the caudate nucleus and NAc, finely modulating the striato‐pallido‐thalamo‐cortical loop (Jentsch and Taylor 1999).

### 7.6 HPA Axis

The intricate interplay between the HPA axis and AUD sheds light on the complex neuroendocrine alterations that underlie the stress response in individuals grappling with AUD (Koob and Volkow 2016).

Evidence points toward HPA axis dysregulation in individuals with AUD, manifesting in augmented cortisol response to stress in those with ongoing alcohol use (Berman et al. 1990; Starcke et al. 2013). However, a study reported no discernible differences in response to stress between control individuals and those with AUD (Bibbey et al. 2015). This paradoxical observation highlights the nuanced and heterogeneous nature of stress reactivity within the AUD population, warranting further exploration to elucidate the factors contributing to this variability.

During acute alcohol withdrawal and exposure to stressors, AUD patients exhibit heightened levels of ACTH and cortisol in comparison to healthy controls, indicating an overactive HPA axis during this vulnerable phase (Costa et al. 1996; Hundt et al. 2001; Muehlhan et al. 2020). This hyperresponsiveness underscores the intricate relationship between alcohol withdrawal, stress, and the dysregulated HPA axis in shaping the neurobiological landscape of AUD.

Contrastingly, in the early abstinence phase (ranging from 2 weeks to 6 months), AUD patients display reduced responsiveness of the adenohypophysis gland to CRH, signifying a deficiency in the stress response mechanism (Inder et al. 1995; Vescovi, DiGennaro, and Coiro 1997). This diminished reactivity may reflect adaptive changes in the HPA axis as individuals navigate the challenging transition from active alcohol use to abstinence.

## 8 Implementation of the Biopsychosocial Model

The biopsychosocial model was developed by Engel (1977) to counteract the biomedical approach in health care. This model is recognized, implemented, and noted within clinical and health education settings while also being the most current holistic framework for contemporary health care (Bolton and Gillett 2019). Essentially, this model endorses a treatment approach considering the biological, psychological, and social components that impact an individual's well‐being. Hunt (2014) describes addiction from a multifactorial perspective by suggesting that addiction has become a multidisciplinary construct that warrants a broad conceptualization from contemporary practitioners. As this understanding evolves, the human service professional must also remain current with this challenging expansion regarding treatment. The findings of this review suggest that the biopsychosocial model would assist clinicians in assessing, treating, and developing plans to match clients’ needs.

Recently, more health‐care professionals, such as psychiatrists and other neuroscientists, have attempted to view addictions as a “brain disease” (Becona 2018). A significant amount of literature from psychological and behavioral scientists has stated that individual behavior is contingent upon several interactive environments, such as the physical, economic, social, and industrial environments (Kickbusch, Allen, and Franz 2016). With this knowledge, treatment for individuals with alcohol addiction and PTSD should focus on a multilayered approach, assessing and evaluating all factors that may impact recovery, or even more concerning, complete abstinence. Within the biopsychosocial model, there are shared themes and concepts that address the etiology and treatment of illness (Bolton 2023). As such, it is imperative that the recognition of biology, neurology, and neuroscience is related to the psychosocial factors in a variety of conditions, in etiology and cause, and in prevention and intervention, these theoretical orientations are comparable to the macro perspective of a biopsychosocial model (Bolton 2023).

## 9 Future Considerations and Recommendations

This review provided a thorough examination of how the brain is affected by PTSD and AUD with a potentially effective treatment approach of the biopsychosocial model. It would be advantageous for addiction researchers to examine the magnetic resonance brain images, computerized axial tomography scans, and positron emission tomography images of individuals with PTSD and AUD before and after applying the biopsychosocial treatment. Such an approach would enhance the comprehension of the nuanced impacts of various treatment strategies, shedding light on their efficacy and potential for fostering neurobiological restoration.

As noted by Bohle, Otterholt, and Bjorkly (2023), implementing the biopsychosocial factors during treatment may have influential clinical implications. When evaluating clients for PTSD and AUD, health practitioners need to be intentional with a comprehensive, holistic approach to better understand what factors are affecting the individual's behavior to use alcohol as a coping mechanism for PTSD. Overall, the biopsychosocial model is a viable, effective, and practical approach to assist individuals with PTSD and AUD while considering the neurobiology underlying both conditions.

## Author Contributions

FH and MM contributed equal percentages to this manuscript. MM primarily wrote the biopyschosocial section, conclusion as FH wrote the neuroscience correlation section and introduction. Both authors have proof read the manuscript and approved.

### Peer Review

The peer review history for this article is available at https://publons.com/publon/10.1002/brb3.70230.

## Data Availability

This was an extensive review of the literature and authors retrieved information from the following databases: PubMed and Google Scholar. We did not collect data for this review; therefore, we do not have a sample size or data inclusion/exclusion criteria to report.
